# Associations between *TNFSF13B* polymorphisms and primary Sjögren's syndrome susceptibility in primary Sjögren's syndrome patients: A meta‐analysis

**DOI:** 10.1002/iid3.1103

**Published:** 2023-12-06

**Authors:** Anhao Zheng, Naiwen Hu, Jing Xu, Ye Yuan, Shumin Zhang, Wenbin Chen, Yanyan Bai, Hongsheng Sun

**Affiliations:** ^1^ Medical Integration and Practice Center Cheeloo College of Medicine, Shandong University Jinan China; ^2^ Department of Rheumatology and Immunology Shandong Provincial Hospital, Cheeloo College of Medicine, Shandong University Jinan China; ^3^ Department of Rheumatology and Immunology Shandong Provincial Hospital, Affiliated to Shandong First Medical University Jinan China; ^4^ Graduate School Shandong First Medical University Jinan China

**Keywords:** B‐cell activating factor, meta‐analysis, polymorphism, Sjögren's syndrome, *TNFSF13B*

## Abstract

**Objective:**

B‐cell activating factor (BAFF) is a key regulator of primary Sjögren's syndrome (pSS), which is characterized by B‐lymphocyte hyperactivity. BAFF, also known as tumor necrosis factor ligand superfamily member 13B, is encoded by *TNFSF13B*. This study aimed to explore the possible relationships between five single‐nucleotide polymorphisms (SNPs) of *TNFSF13B* (rs9514827, rs1041569, rs9514828, rs1224141, and rs12583006) and pSS susceptibility.

**Methods:**

We searched the following databases for articles on *TNFSF13B* polymorphism and pSS published up to January 2023: PubMed, Cochrane, Elsevier, Web of Science, CNKI, CQVIP, and WanFang. The odds ratios (with 95% confidence intervals) of genotypes and SNP alleles of *TNFSF13B* were investigated in patients with pSS to determine their relationships with pSS.

**Results:**

This meta‐analysis employing the fixed‐effect model comprised three studies of pSS patients and randomly selected healthy controls (HCs), revealing statistically significant relationships between pSS susceptibility and two SNPs: rs1041569 and rs12583006. Because rs1041569 was not in Hardy‐Weinberg equilibrium in the HC group, it was eliminated from the analysis.

**Conclusions:**

Polymorphisms in the BAFF (*TNFSF13B*) gene were related to vulnerability to pSS among pSS patients and HCs alike. The SNP rs12583006 was significantly related to pSS susceptibility in pSS patients.

## Graphical abstract

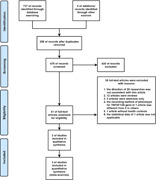

## 1 INTRODUCTION

Sjogren's syndrome (SS) is a chronic autoimmune disorder characterized by lymphocytic infiltration of exocrine glands, most notably the salivary and lacrimal glands, resulting in oral and ocular dryness. In addition to clinical functional impairment of the salivary and lacrimal glands, multi‐system and multiorgan involvement may occur, with autoantibodies and hyperimmune globulinemia in the serum. SS is divided into two types based on accompaniment by other connective tissue diseases: secondary SS and primary SS (pSS). The former often develops secondary to systemic lupus erythematosus, rheumatoid arthritis (RA), or other autoimmune diseases. The syndrome often has an insidious onset, with clinical manifestations that vary in severity. Therefore, most patients do not notice their symptoms, delaying diagnosis and treatment and resulting in serious clinical manifestations, such as rampant dental caries and interstitial pneumonia, which can lower their quality of life. SS is identified by B‐cell hyperactivity characterized by hypergammaglobulinemia and a variety of autoantibodies, with B‐cell activating factor (BAFF; also known as BLyS), a B‐cell survival factor, playing a significant role.^1^ However, the pathogenesis and etiology of pSS remain unclear. The presence of anti‐Ro/SSA autoantibodies has emerged as one of the most well‐established and highly positively correlated risk factors for pSS. Whereas anti‐Ro/SSA autoantibodies lack specificity for SS, many studies have shown that high expression of the BAFF coding gene, tumor necrosis factor (TNF) ligand superfamily member 13B (*TNFSF13B*), is also involved in pSS.^1^, ^2^, ^3^, ^4^, ^5^, ^6^, ^7^, ^8^, ^9^, ^10^, ^11^, ^12^, ^13^, ^14^, ^15^, ^16^, ^17^, ^18^, ^19^, ^20^, ^21^, ^22^, ^23^, ^24^, ^25^, ^26^, ^27^, ^28^, ^29^, ^30^, ^31^, ^32^, ^33^, ^34^, ^35^, ^36^ TNFSF13B/BAFF is a cytokine that belongs to the TNF ligand family. BAFF aids B‐cell proliferation, maturation, differentiation, and immunoglobulin synthesis.^2^ Various single‐nucleotide polymorphisms (SNPs) in *TNFSF13B* have been linked to vulnerability to various autoimmune diseases.^8^

Previous research has suggested that the genotype and allelic polymorphisms of *TNFSF13B* are involved in pSS. One study showed that the same genotype influences pSS vulnerability in both the codominant and recessive models. Specifically, the TTTAC haplotype was discovered to enhance pSS susceptibility.^2^ Another study showed that *TNFSF13B* mRNA expression was elevated by 2.43‐fold and 5.04‐fold in patients with RA and pSS compared with healthy controls (HCs), respectively.^3^ The findings of a third study suggested that the CTAT haplotype increases disease susceptibility for Ro/La‐positive pSS, but is not linked with high serum BAFF (s‐BAFF) levels. Elevated s‐BAFF levels in pSS are linked to the TTTT genotype and could be a secondary effect of Ro/La‐positive pSS.^25^ In addition, research has shown that distinct haplotypes of *TNFSF13B* confer greater susceptibility to pSS development, highlighting the importance of genetic polymorphisms in *TNFSF13B* in the development of pSS as well as pSS‐related lymphomagenesis.^1^

Although the authors of one study concluded that both a genetic propensity for pSS and a specific pattern of antibody production are unrelated to the polymorphisms in *TNFSF13B*, they only examined one SNP: −871 T/C.^37^ Therefore, in this work, we conducted the first meta‐analysis of data on the relationship between pSS susceptibility and *TNFSF13B* polymorphisms. The purpose of our study was to investigate the pathogenesis of pSS, with a focus on *TNFSF13B* polymorphisms as an underlying cause of the elevated levels of serum autoantibodies in pSS. We anticipate that our findings will contribute to the development of more precise methods for the clinical diagnosis of pSS. Early diagnosis will enable pSS patients to receive timely treatment, potentially stabilizing or slowing progression of the disease.

## 2 MATERIALS AND METHODS

### 2.1 Literature collection strategy

Databases PubMed, Cochrane, Embase, Web of Science, CNKI, WanFangData, and Cqvip were searched for publications in any language, from any location, from the start date of each database through January 2023. Searches were conducted using the terms “Sjögren's syndrome,” “TNFSF13B,” and “BAFF,” along with associated nouns. To find other studies that were not indexed by our initial search, the reference lists of pertinent research articles were also examined. Full‐text articles were retrieved from the Shandong University library, or obtained from the author, as necessary.

### 2.2 Inclusion and exclusion criteria

Qualifying studies had to satisfy each of the following criteria for inclusion: (1) case‐control study design; (2) the association between pSS and *TNFSF13B* polymorphisms was examined; (3) information on the frequencies of *TNFSF13B* genotypes and alleles for both pSS patients and HCs was provided; and (4) study subjects were positive for *TNFSF13B* expression. The following were excluded from the meta‐analysis: (1) duplicate data found in multiple studies; (2) research that was completely irrelevant to the theme of this meta‐analysis, such as mice experiment; (3) research conducted on a family; (4) research direction that was inconsistent with this meta‐analysis, such as serum BAFF concentration; (5) review articles; (6) abstract‐only publications; (7) research using a recording method for *TNFSF13B* phenotype that was different from this meta‐analysis so that harmonized calculations are not possible, such as −871 T/C; (8) studies without HCs; and (9) inapplicable statistical data.

### 2.3 Data extraction

The first author, publication year, ethnicity of the research population, numbers of cases and controls, and the frequencies of *TNFSF13B* genotypes and alleles were all independently acquired from each article by two reviewers.

### 2.4 Methodological quality assessment

Using the Newcastle‐Ottawa Scale (NOS) of 0–9 stars, two reviewers independently evaluated the methodological quality of each included study. Studies that received ≥6 stars were regarded as being of high quality.

### 2.5 Statistical analysis

Statistical analysis was conducted using STATA 16.0 (Stata Corp LP). *TNFSF13B* polymorphisms and pSS susceptibility were shown to be associated using odds ratios (ORs) and 95% confidence intervals (CIs). The STATA16.0 method was used to compute the Hardy‐Weinberg equilibrium (HWE) of each SNP, with a cutoff of *p* ＜ .05 indicating HWE divergence. The fixed‐effects and random‐effects models were used to obtain the pooled ORs. I^2^ statistics were used to identify heterogeneity between studies. When heterogeneity was significant, the random‐effects model was chosen. The fixed‐effects model was appropriate for I^2^ statistics at I^2^ ＜ 50%; otherwise, the random‐effects model was chosen. Funnel plots were used to evaluate publication bias. All statistical tests used in this study were two‐sided, and a study was deemed statistically significant at a *p* ＜ .05.

## 3 RESULTS

### 3.1 Literature search and study characteristics

**Figure 1:**
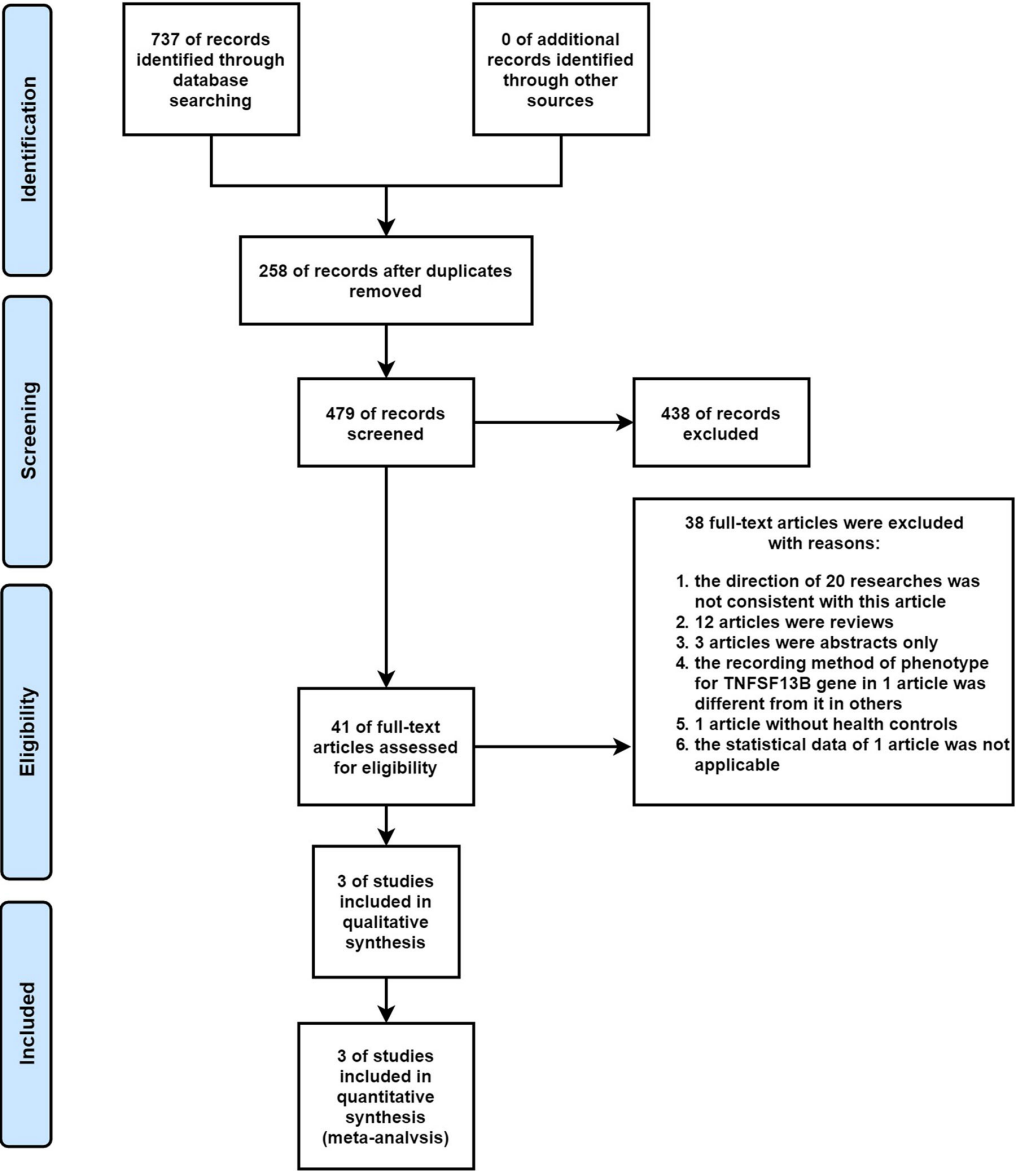
The flow chart of search results.

Our initial search generated 737 items that satisfied the search criteria. Other sources did not produce additional research. Overall, 258 articles containing duplicate content were removed. An additional 438 articles that did not meet our criteria were also removed. The remaining 41 articles were thoroughly assessed, of which 38 were excluded for a variety of reasons. Ultimately, three high‐quality case‐control studies from three articles were examined here (Figure 1).

The relevant studies were published between 2014 and 2023. In one, the sample size ranged from 101 to 193 for pSS patients and 137–309 for HCs. Athenian, Mexican, and Caucasian Greek ethnicities were represented in the three studies. The meta‐analysis comprised 646 HCs and 442 pSS patients in whom *TNFSF13B* SNPs were studied. The genotypic frequencies of SNPs in the HC group were consistent with HWE (*p* ＞ .05). Table 1 shows the features of the pSS patients and HCs in the three eligible studies.

**Table 1:** Basic information on the pSS patients and HC subjects included in the meta‐analysis.

Year	First author	Country	Ethnicity	pSS No	HC No	SNPs	HWE
2023	Nikolaos Kintrilis	Athens	Athenian	148	200	rs9514827	Yes
						rs1041569	
						rs9514828	
						rs1224141	
						rs12583006	
2022	Enrique Santillán‐ López	Mexico	Mexican	101	309	rs9514827	Yes
						rs1041569	
						rs9514828	
2014	Adrianos Nezos	Greek	Caucasian	193	137	rs9514827	Yes
						rs1041569	(Except rs1041569)
						rs9514828	
						rs1224141	
						rs12583006	

Abbreviations: HC, healthy controls; HWE, Hardy–Weinberg equilibrium; pSS, primary Sjögren's syndrome patients; SNP, single‐nucleotide polymorphism.

### 3.2 Relationship between rs9514827 and pSS susceptibility in random HCs and pSS patients

**Figure 2:**
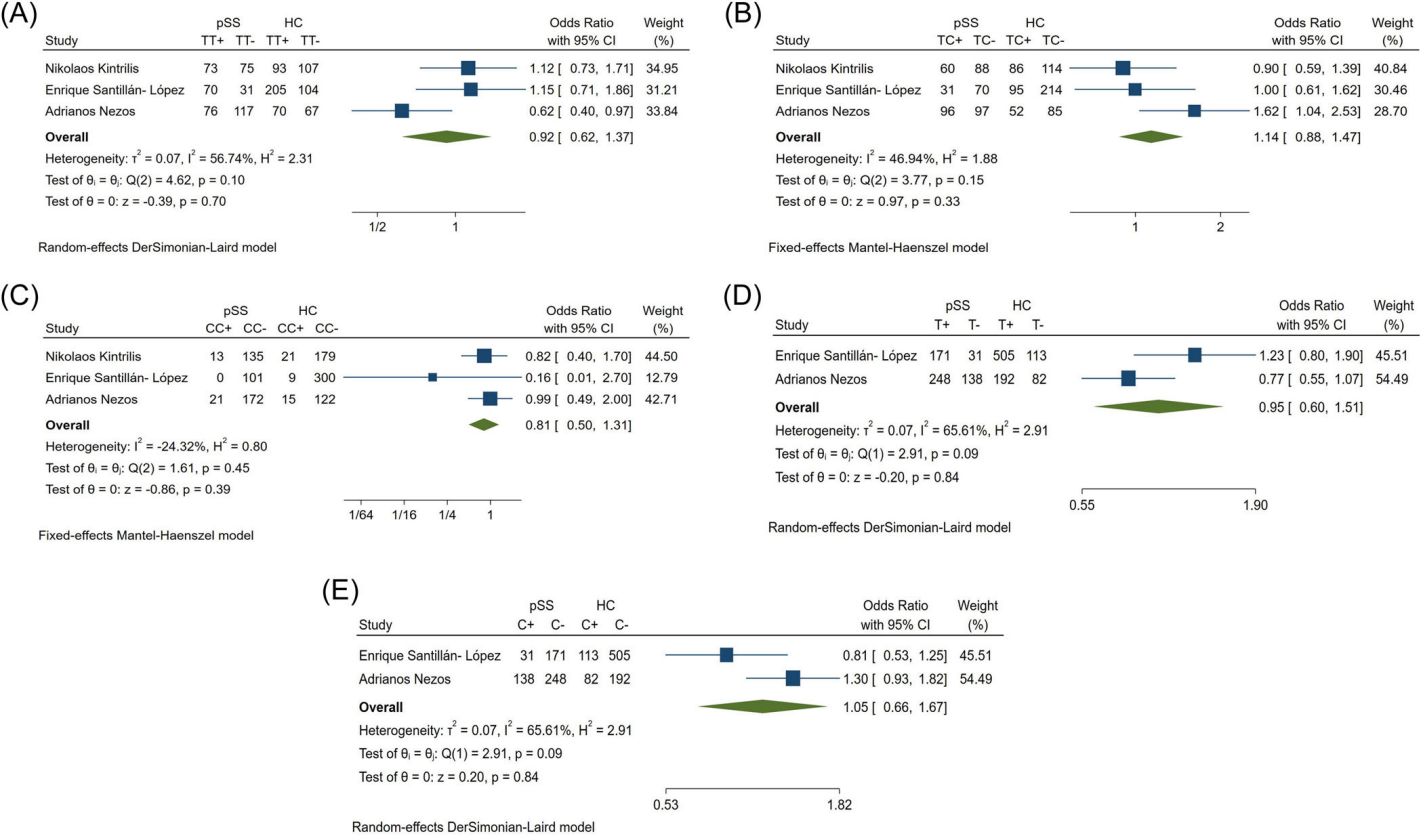
Forest plots of rs9514827 (A) TT; (B) TC; (C) CC; (D) T; (E) C; between pSS patients and healthy controls. HC, healthy controls; pSS, primary Sjogren's syndrome patients.

There were no statistical differences in rs9514827 frequency between pSS patients and HCs (Table 2). On the basis of the heterogeneity (Figure 2A–E), the TT and CC genotypes were analyzed using the fixed model, and the TC genotype and the T and C alleles were analyzed using the random model. The ORs, 95% CIs, and *p* values for each genotype and allele of this SNP are shown in Table 2. There were no relationships between rs9514827 and pSS susceptibility (Table 2, Figure 2A–E).

**Table 2:** Summary of ORs (95%CI) in the analysis of the relationship between *TNFSF13B* and pSS susceptibility in pSS patients and HCs.

Comparison	Study (*n*)	Genotype/Allele	Fixed effects		Random effects		Heterogeneity			Publication bias	
			OR (95% CI)	*p*	OR (95% CI)	*p*	Q	*p*	I^2^ (%)	Egger's test	*p*
rs9514827	3	TT	1.14 (0.88–1.47)	.33	0.92 (0.62–1.37)	.70	4.62	0.10	56.74	0.27	.79
		TC					3.77	0.15	46.94	−0.11	.91
		CC					1.61	0.45	−24.32	−1.20	.23
		T			0.95 (0.60–1.51)	.84	2.91	0.09	65.61	0.88	.38
		C	0.81 (0.50–1.31)	.39	1.05 (0.66–1.67)	.84	2.91	0.09	65.61	−0.88	.38
rs1041569	3	AA	0.96 (0.74–1.26)	.78			0.24	0.88	−718.34	0.34	.74
		AT	0.91 (0.70–1.20)	.52			0.12	0.94	−1592.91	0.16	.88
		TT	4.62 (1.59–13.43)	.00			2.11	0.35	5.43	−0.12	.90
		A	0.95 (0.70–1.27)	.72			0.15	0.69	−545.45	0.39	.69
		T	1.06 (0.78–1.42)	.72			0.15	0.69	−545.45	−0.39	.69
rs9514828	3	CC			0.99 (0.63–1.56)	.98	4.69	0.10	57.36	−2.10	.28
		CT	0.83 (0.64–1.07)	.15			1.94	0.38	−2.99	−0.12	.91
		TT	1.33 (0.94–1.87)	.10			0.03	0.99	−7819.51	0.10	.92
		C	0.88 (0.70–1.12)	.31			1.81	0.18	44.84	1.35	.18
		T	1.13 (0.89–1.43)	.31			1.81	0.18	44.84	−1.35	.18
rs1224141	2	TT	0.75 (0.53–1.05)	.09			0.00	0.95	−27360.62	−0.06	.95
		GT	1.24 (0.88–1.75)	.22			0.08	0.78	−1229.40	−0.27	.78
		GG	2.32 (0.77–7.00)	.14			1.10	0.30	8.74	1.03	.31
rs12583006	2	TT	0.73 (0.54–0.99)	.04			1.76	0.18	43.21	−1.33	.18
		TA	1.08 (0.79–1.49)	.63			1.04	0.31	3.43	1.02	.31
		AA	2.55 (1.34–4.86)	.00			0.01	0.90	−6574.89	−0.12	.90

### 3.3 Relationship between rs1041569 and pSS susceptibility in random HCs and pSS patients

**Figure 3:**
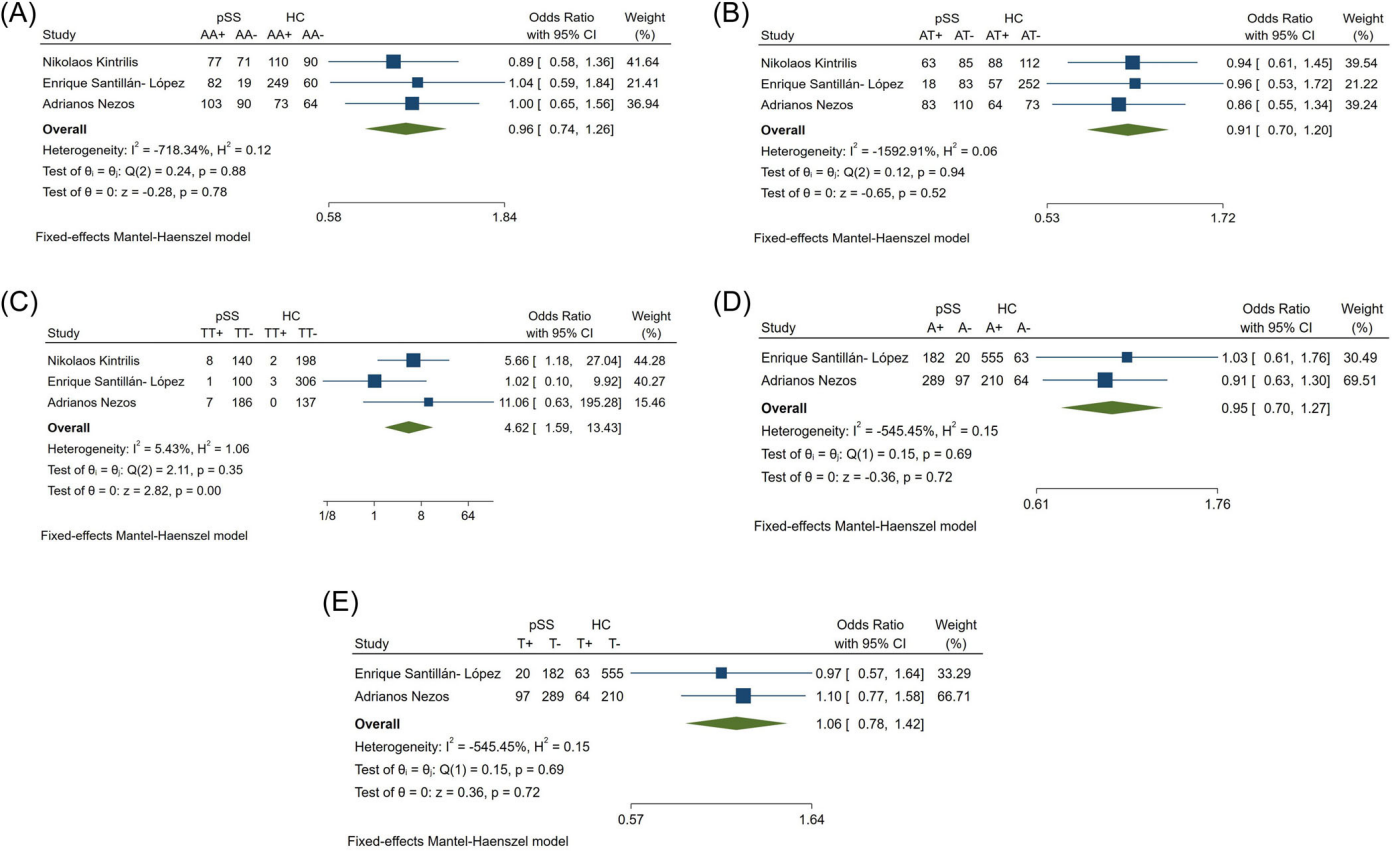
Forest plots of rs1041569 (A) AA; (B) AT; (C) TT; (D) A; (E) T; between pSS patients and healthy controls. HC, healthy controls; pSS, primary Sjogren's syndrome patients.

There was a statistical difference in rs1041569 frequency between pSS patients and HCs (Table 2). On the basis of the heterogeneity (Figure 3A–E), the AA, AT, and TT genotypes, and the A and T alleles were analyzed using the fixed model, with ORs, 95% CIs, and p values shown in Table 2. There was a relationship between the TT genotype of rs1041569 and pSS susceptibility (Table 2, Figure 3A–E). The rs1041569 results were eliminated because the frequency of this SNP did not comply with HWE in the HC group.

### 3.4 Relationship between rs9514828 and pSS susceptibility in random HCs and pSS patients

**Figure 4:**
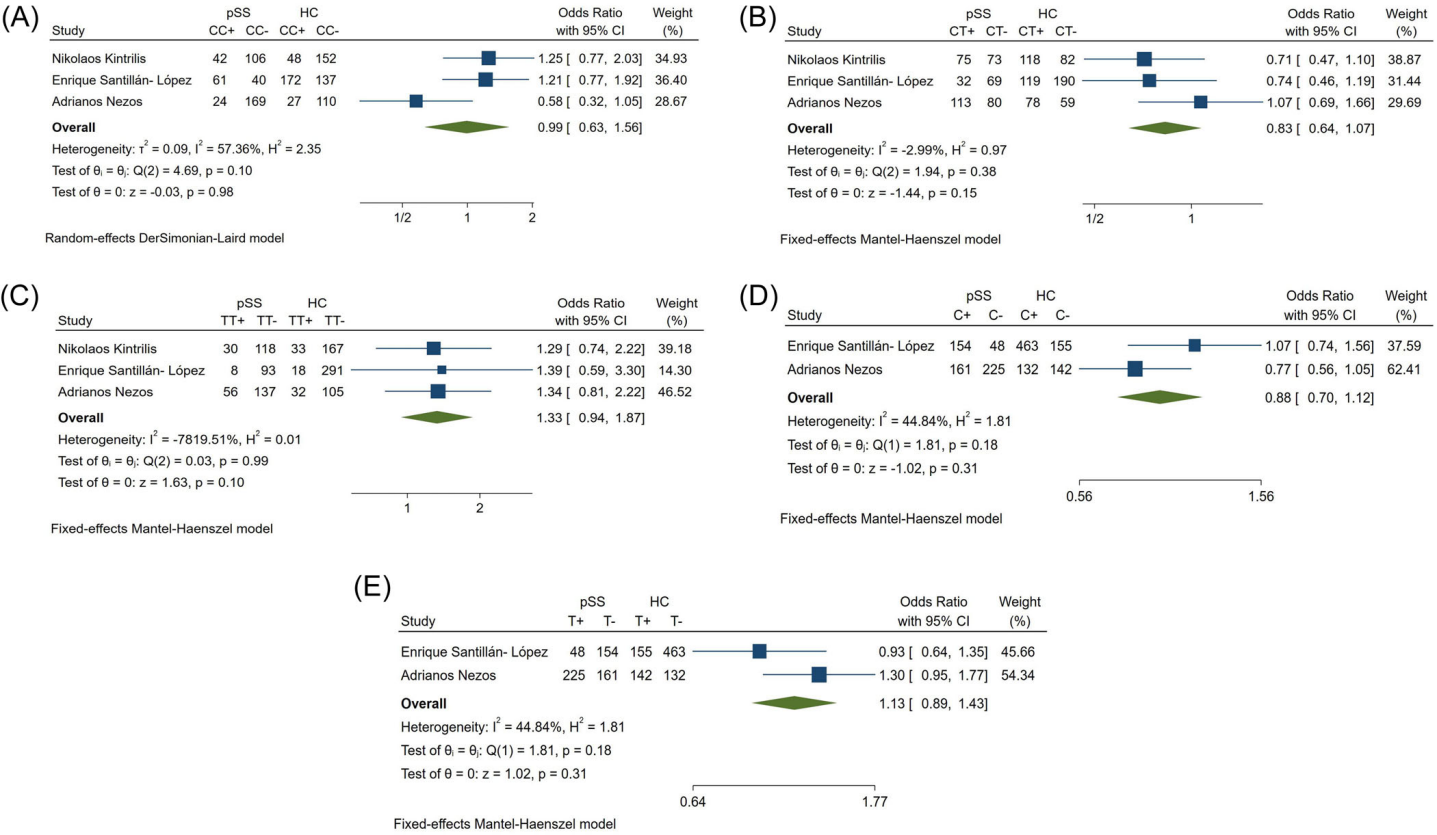
Forest plots of rs9514828 (A) CC; (B) CT; (C) TT; (D) C; (E) T; between pSS patients and healthy controls. HC, healthy controls; pSS, primary Sjogren's syndrome patients.

There were no statistical differences in rs9514828 frequency between pSS patients and HCs (Table 2). On the basis of the heterogeneity (Figure 4A–E), the CT and TT genotypes and the C and T alleles were analyzed using the fixed model, and the CC genotype was analyzed using the random model, with ORs, 95% CIs, and p values for each genotype and allele shown in Table 2. There was no relationship between rs9514828 and pSS susceptibility.

### 3.5 Relationship between rs1224141 and pSS susceptibility in random HCs and pSS patients

**Figure 5:**
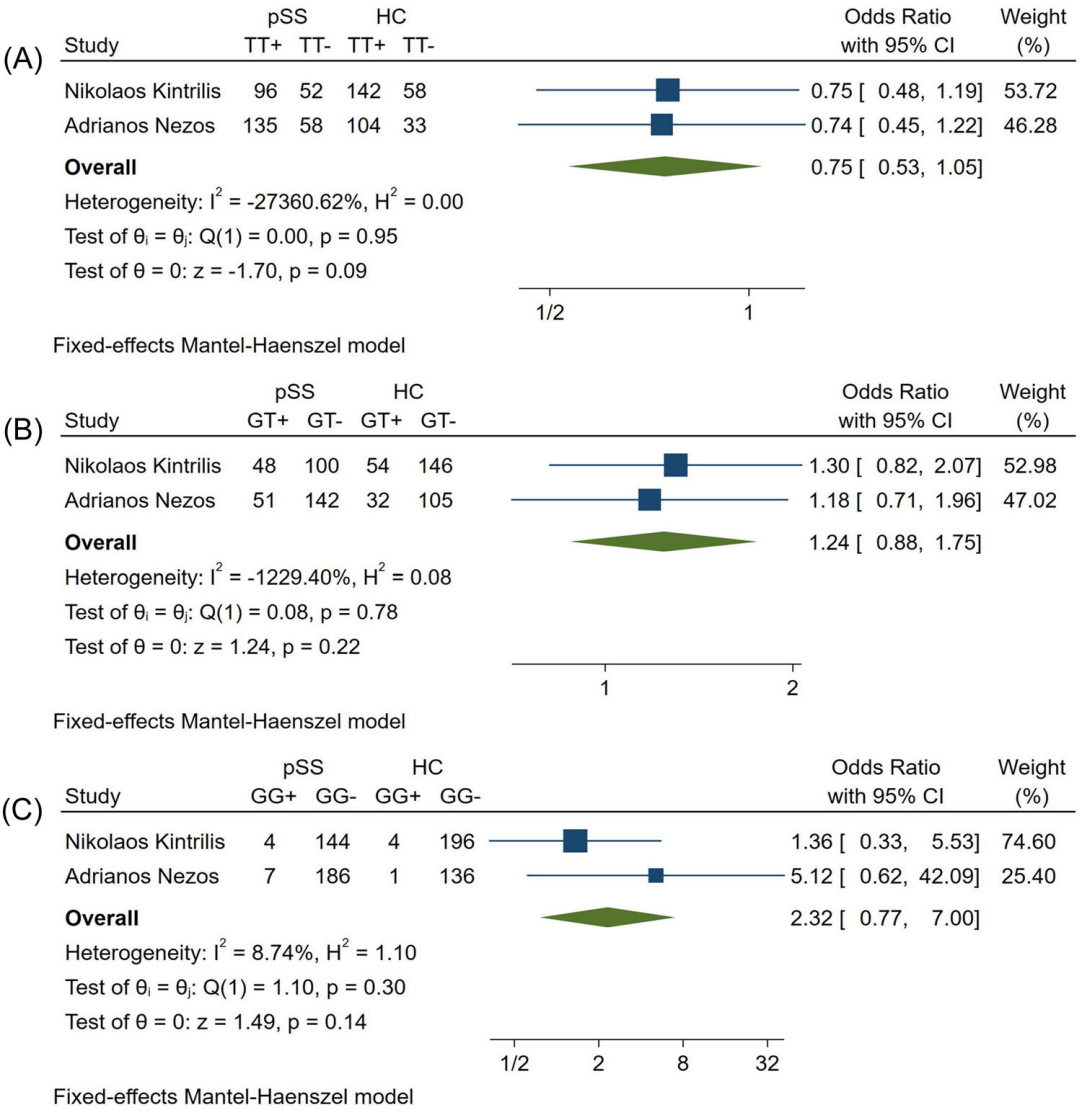
Forest plots of rs1224141 (A) TT; (B) GT; (C) GG; between pSS patients and healthy controls. HC, healthy controls; pSS, primary Sjogren's syndrome patients.

There were no statistical differences in rs1224141 frequency between pSS patients and HCs (Table 2). On the basis of the heterogeneity (Figure 5A–C), the GG, GT, and TT genotypes were analyzed using the fixed model, with ORs, 95% CIs, and p values for each genotype and allele shown in Table 2. There was no relationship between rs1224141 and pSS susceptibility (Table 2, Figure 5A–C).

### 3.6 Relationship between rs12583006 and pSS susceptibility in random HCs and pSS patients

**Figure 6:**
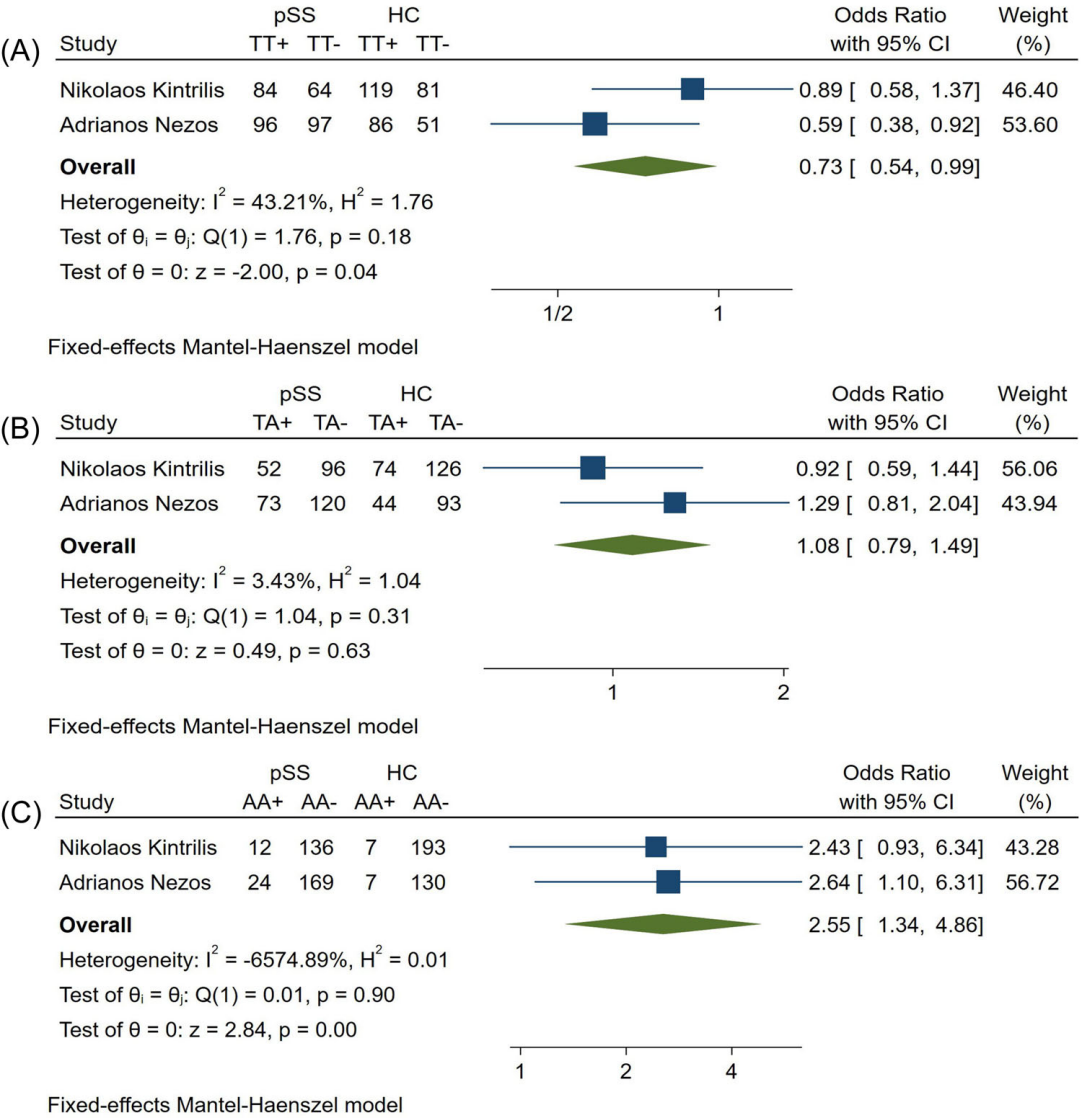
Forest plots of rs12583006 (A): TT; (B) TA; (C) AA; between pSS patients and healthy controls. HC, healthy controls; pSS, primary Sjogren's syndrome patients.

There were statistical differences in rs12583006 frequency between pSS patients and HCs (Table 2). On the basis of the heterogeneity (Figure 6A–C), the AA, TA, and TT genotypes were analyzed using the fixed model, with ORs 95% CIs, and p values for each genotype and allele shown in Table 2. There were relationships between pSS susceptibility and the AA and TT genotypes, but not the TA genotype, of rs12583006 (Table 2, Figure 6A–C).

### 3.7 Publication bias

Using Egger's test, we found no discernible publication bias for any of the genotypes or alleles of SNPs rs9514827, rs1041569, rs9514828, rs1224141, and rs12583006 (Table 2).

## 4 DISCUSSION

Numerous studies on pSS and *TNFSF13B* have mostly focused on five SNPs: rs9514828, rs1041569, rs9514827, rs12583006, and rs1224141. Most of these SNPs have been previously associated with increased susceptibility to pSS, but with subtle differences in opinion of the study authors. Kintrilis et al. concluded that genotype TT of rs1041569, a variant of *TNFSF13B*, had a significantly increased prevalence in pSS patients compared to that in HCs.^2^ They also demonstrated that genotype TT of rs1041569 was a risk factor for pSS, altering susceptibility to pSS, and that haplotype TTAC also increased susceptibility to pSS. These authors also reported that haplotypes TATTT and TTCTT were only detected in pSS patients with thickened arterial walls.^2^ Santillan‐Lopez et al., demonstrated that the expression of *TNFSF13B* mRNA was increased by 5.04‐fold in pSS patients compared to that in HCs. They also investigated the relationship between soluble BAFF and *TNFSF13B* expression and found that elevated *TNFSF13B* expression levels were consistent with soluble BAFF protein levels. However, they concluded that *TNFSF13B* transcript levels in pSS patients were not associated with the SNP rs9514828.^3^ Nezos et al. concluded that the *TNFSF13B* polymorphism increased susceptibility to pSS in both high‐risk (type I) pSS patients and low‐risk (type II) pSS patients compared to that in HCs.^1^ In their high‐risk group of pSS patients, the frequencies of the CC genotype of rs9514828 and the TT genotype of rs9514827 were statistically different from those of HCs. In their low‐risk group of pSS patients, the frequencies of the minor A allele and the AA genotype of rs12583006 were statistically different from those of HCs.^1^ Another study identified the TT genotype of rs9514828 as a protective factor against fatigue in patients with pSS.^38^

The results of our meta‐analysis demonstrated that the TT genotype of rs1041569 and the AA and TT genotypes of rs12583006 all statistically significantly increase the susceptibility to pSS. Lack of HWE in the HC group necessitated that we eliminate the results of rs1041569 analysis. Furthermore, our results suggested that the AA genotype of rs12583006 is a risk factor (OR: 2.55 [CI: 1.34–4.86], *p* = .00 in the fixed‐effects model), whereas the TT genotype of rs12583006 is a protective factor (OR: 0.73 [CI: 0.54‐0.99], *p* = .04 in the fixed‐effects model).

As research has progressed, the role of TNFSF13B/BAFF in pSS has been explored. Nossent et al. suggested that susceptibility to Ro/La‐positive pSS is increased with the CTAT haplotype and that elevated s‐BAFF levels in pSS patients are associated with the TTTT haplotype.^25^ Carrillo‐Ballesteros et al. concluded that pSS patients with elevated s‐BAFF levels have longer disease duration and the highest levels of anti‐La/SSB antibodies. Additionally, they found that the duration of the disease was associated with anti‐Ro/SSA antibodies and SS disease activity index scores.^6^ Loureiro‐Amigo et al. determined that serum levels of BAFF, CXCL13, and PD‐L2 showed the highest accuracy in identifying patients with pSS, with significant differences between patients and controls.^5^ They found that the highest diagnostic accuracy was obtained by applying serum levels of BAFF, CXCL13, and PD‐L2 to the formula [ln(CXCL13) + ln(BAFF)]/ln(PD‐L2), resulting in an area under the curve value of 0.854, with a sensitivity of 77.2% and specificity of 86.4%, using a cut‐off value of 1.7.^5^ Another study showed that eight of nine optimal immune‐related genes (IRGs; *IL‐18*, *JAK2*, *TBK1*, *EED*, *TNFSF10*, *TNFSF13B*, *CYSLTR1*, and *ICOS*) were significantly overexpressed in pSS patients compared with that in HCs.^4^ Using quantitative reverse transcription PCR, these IRGs were defined as key genes in the occurrence and development of pSS. Furthermore, receiver operating characteristic analysis showed high sensitivity and specificity for *TNFSF13B* and *CYSLTR1*.^4^

In conclusion, our meta‐analysis of published data demonstrated that polymorphisms in *TNFSF13B* were related to vulnerability to pSS among pSS patients and HCs. The respective ORs and 95%CIs revealed that the AA genotype of rs12583006 is a risk factor in pSS patients, and the TT genotype of rs12583006 is a protective factor in pSS patients. Our analysis indicates that *TNFSF13B* is associated with susceptibility to pSS and s‐BAFF levels, which are associated with B‐cell activation and autoantibody production. Additionally, increased anti‐Ro/La antibody levels were associated with increased s‐BAFF levels. We speculate that activation of *TNFSF13B*, especially via the SNP rs12583006, increases s‐BAFF levels, thereby enhancing B‐cell activation and increasing autoantibody production, leading to the occurrence and development of pSS. Therefore, the detection of *TNFSF13B* polymorphism, especially the TT and AA genotypes of rs12583006, will provide more direction for the diagnosis of pSS. Earlier clinical diagnosis of suspected pSS could lead to earlier treatment, delaying or stabilizing the progression of pSS, improving quality of life, and alleviating the anxious mental state of pSS patients. Further investigation into the relationships between TNFSF13B, s‐BAFF levels, and autoantibody levels, and their sensitivity and specificity, may shed light on the process by which genetic polymorphisms affect the production of autoantibodies in patients with pSS, aiding in clinical diagnosis and therapy. In the future, treatment targeting *TNFSF13B* may lead to a cure for pSS.

## AUTHOR CONTRIBUTIONS

**Anhao Zheng**: Conceptualization; Data curation; Formal analysis; Methodology; Resources; Software; Visualization; Writing—original draft. **Naiwen Hu**: Supervision. **Jing Xu**: Data curation. **Ye Yuan**: Data curation. **Shumin Zhang**: Data curation. **Wenbin Chen**: Funding acquisition. **Yanyan Bai**: Investigation; Methodology; Validation; Writing—review & editing. **Hongsheng Sun**: Project administration; Resources; Validation; Writing—review & editing.

## CONFLICT OF INTEREST STATEMENT

The authors declare no conflict of interest.

## Data Availability

The authors have nothing to report.
